# The Role of *Flavobacterium enshiense* R6S-5-6 in the Wetland Ecosystem Revealed by Whole-Genome Analysis

**DOI:** 10.1007/s00284-022-03157-0

**Published:** 2023-01-21

**Authors:** Ahhyeon Choi, In-Tae Cha, Ki-Eun Lee, Youn Kyoung Son, Jaewoong Yu, Donghyeok Seol

**Affiliations:** 1 eGnome, Inc, 26 Beobwon-ro 9-Gil, Songpa-gu, Seoul, 05836 Republic of Korea; 2 National Institute of Biological Resources, 42 Hwangyeong-ro, Seo-gu, Incheon, 22689 Republic of Korea; 3 Department of Agricultural Biotechnology and Research Institute of Agriculture and Life Sciences, Seoul National University, 1 Gwanak-ro, Gwanak-gu, Seoul, 08826 Republic of Korea; 4 Present Address: Department of Surgery, Seoul National University Bundang Hospital, 172 Dolma-ro, Bundang-gu, Seongnam, 13605 Republic of Korea

## Abstract

The wetland is an important ecosystem for purifying pollutants and circulating nutrients. Numerous microorganisms contribute to maintaining this function. We obtained *Flavobacterium enshiense* R6S-5-6 which was isolated from Ungok (Ramsar) Wetland and conducted whole-genome sequencing to investigate what contribution R6S-5-6 could make to the wetland community. The complete genome sequence of R6S-5-6 has a size of 3,251,289 bp with 37.68% of GC content. Gene annotation revealed that R6S-5-6 has several pathways to break down pollutants, including denitrification, assimilatory sulfate reduction (ASR), and polyphosphate-accumulating process. Furthermore, R6S-5-6 has genes that can have a positive effect on plants living in wetlands, such as storing essential nutrients, promoting plant growth, and protecting plants against pathogens.

**Supplementary Information:**

The online version contains supplementary material available at 10.1007/s00284-022-03157-0.

## Introduction

Wetlands are among the most productive ecosystems on the planet. They are often referred to as “Earth’s kidneys” because they play a crucial role in managing greenhouse gas emissions, controlling floods and droughts, and purifying pollutants from water [1]. Also, they usually provide a nutrient-rich habitat for living things, which results in unique biodiversity [2]. In particular, the members of the wetlands bacterial ecosystem include sulfate-reducing, nitrogen-fixing, denitrifying, and methanotrophic bacteria [3]. These bacteria are involved in several biogeochemical cycles, including carbon, nitrogen, and sulfur, affecting vegetation ecology [4].

The genus *Flavobacterium*, a member of the family *Flavobacteriaceae*, was first proposed by *Bergey* et al. [5]. *Flavobacterium* is widely distributed in the environment, including activated sludge [6], stream sediment [7], glacier [8], and rhizosphere [9]. *Flavobacterium* not only has a wide range of habitats but also has a wide range of characteristics, such as pathogenicity, which causes fatal damage to both wild and cultured fish, and plant growth-promoting properties [10, 11].

*Flavobacterium enshiense* was first reported by *Dong* et al. in 2013 and consists of only one type strain, designated DK69^T^ [12]. *F. enshiense* DK69^T^ is a Gram-negative, strictly aerobic, yellow-pigmented, and rod-shaped bacterium isolated from soil collected from the wastewater treatment plant in Enshi, Hubei province, China. In the chemotaxonomic data, *F. enshiense* DK69^T^ contained menaquinone-6 and phosphatidylethanolamine as the major quinone and polar lipid, respectively. In the whole-genome analysis of *F. enshiense* DK69^T^, no pathogenic genes were found [13].

Here, we report the complete genome of a novel strain of *F. enshiense*, designated R6S-5-6, for a better understanding of the species *F. enshiense* and bacteria in wetlands. The complete genome sequence of R6S-5-6 is expected to broaden our understanding of the genomic features of *F. enshiense* and the characteristics of wetland bacteria.

## Materials and Methods

### Sample Preparation and Genome Sequencing

From the National Institute of Biological Resources Culture Collection (NIBR), we obtained the strain R6S-5-6 which was isolated from Ungok (Ramsar) Wetland located in Gochang, Jeollabuk-do province, the Republic of Korea in 2011 (NIBRBA0000113315). According to the sample details provided, a wetland sample from Gochang was preserved in a 50-ml conical tube in 2011. The sample was serially diluted and plated onto R2A agar. After inoculation, appeared colonies were moved to the same fresh medium at 25 ºC. A purified single colony was confirmed with PCR amplification using universal bacterial 16S rRNA gene primers 27F and 1492R (GenBank accession: JQ928689). The selected colony was termed strain R6S-5-6. The strain was preserved at − 80 ºC until conducting whole-genome sequencing.

A genomic DNA was extracted using RBC DNA extraction kit according to the manufacturer instruction. The genomic DNA was purified, sheared, and normalized for PacBio SMRTbell 20 kb Library [14]. Continuous long reads (CLR) were produced by sequencing the SMRTbell library onto the PacBio Sequel platform.

### Genome Assembly

We used Samtools (v1.13) to convert PacBio BAM file to FASTA and removed short reads under 1000 bp that could affect assembly quality. De novo genome assembly was conducted using Flye (v2.8.3) with –meta option as suggested to be effective for controlling any contamination or artifacts. After excluding contigs of less than 10,000 bp that were suspected of contamination or artifacts, the errors of long circular contig were polished using pbmm2 (v1.4.0) and GCpp (2.0.2) with Arrow algorithm until no more variants were identified (https://github.com/PacificBiosciences/GenomicConsensus). The *dnaA* gene in the genome was rotated to the starting point using Circlator (v1.5.5). CheckM (v1.1.3) and BUSCO (v5.2.2) with flavobacteriales_odb10 dataset were used to evaluate the quality of assembly.

### Phylogenetic Analysis

Phylogenetic analysis of R6S-5-6 was carried out using 16S rRNA gene, *gyrB* gene, and orthologs. The 16S rRNA gene and whole-genome sequence for type strain of *Flavobacterium* spp. were retrieved from GenBank [15]. For 16S rRNA gene, sequences were aligned using SINA Aligner (v1.2.11) and trimmed using trimAl (v1.4.rev15) with ‘-gappyout’ option. The sequences of the *gyrB* gene and single-copy orthologs were obtained using Prokka (v1.14.6) and OrthoFinder (v2.5.4). Multiple sequence alignment for *gyrB* and orthologs was performed using MAFFT-linsi (v7.487), and they were trimmed using ClipKIT (v.1.3.0) with ‘kpic-smart-gap’ mode. All of alignments were used for a maximum-likelihood tree construction using IQTREE (v2.1.4 beta) under model testing with 1000 ultrafast bootstraps. Protein sequence identity for *gyrB* gene was calculated through VectorBuilder’s Sequence Alignment tool (https://www.vectorbuilder.com/tool/sequence-alignment.html). Average nucleotide identity (ANI) and digital DNA-DNA hybridization (dDDH) values were calculated using pyani (v0.2.12) and Genome to Genome Distance Calculator (GGDC; v3.0), respectively. For further confirmation of the taxonomic assignment, the whole-genome was submitted to the Type Strain Genome Server (TYGS), the Microbial Genome Atlas (MiGA), and the fast bacterial genome identification platform (fIDBAC). Whole-genome-based taxonomic assignment was also conducted using GTDB-Tk (v1.6.0).

### Genome Annotation

Functional annotation was carried out using Prokka, Rapid Annotation using Subsystems Technology (RAST) server, eggNOG-mapper (v2.1.6) with eggNOG 5.0 database, and MicrobeAnnotator (v2.0.5). Other genomic features were investigated using dbCAN2 for Carbohydrate-Active enZYmes (CAZymes), PHASTER for prophage sequences, and IslandViewer4 for genomic islands. Secondary metabolite biosynthesis gene clusters were identified using antiSMASH (v6.0) with “loose” detection strictness. Operons and clustered regularly interspaced short palindromic repeats (CRISPRs) were predicted with Operon-mapper and CRISPRCasFinder, respectively. Enzyme commission number (EC number) was predicted by DeepEC (v0.4.0). Metabolic pathway of enzymes was visualized by iPATH3. Virulence genes were identified using ABRicate (v1.0.1) (https://github.com/tseemann/abricate) with VFDB.

## Results

### Genome Features of R6S-5-6

**Fig. 1:**
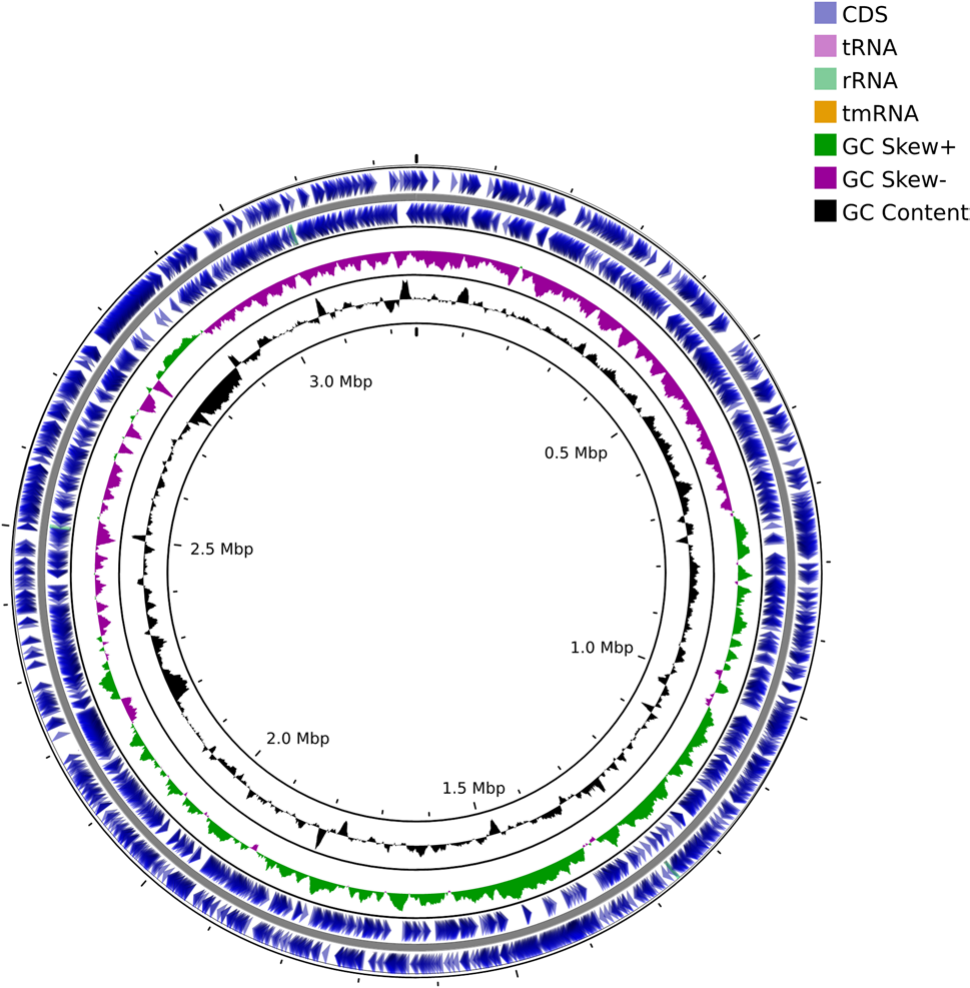
Complete genome map of R6S-5-6. From outside to the center: CDS (blue), tRNA (lavender), rRNA (jade), tmRNA (yellow), GC Skew + (green), GC Skew- (purple), and GC Content(black). The map was generated using CGView Server BETA (Color figure online)

**Fig. 2:**
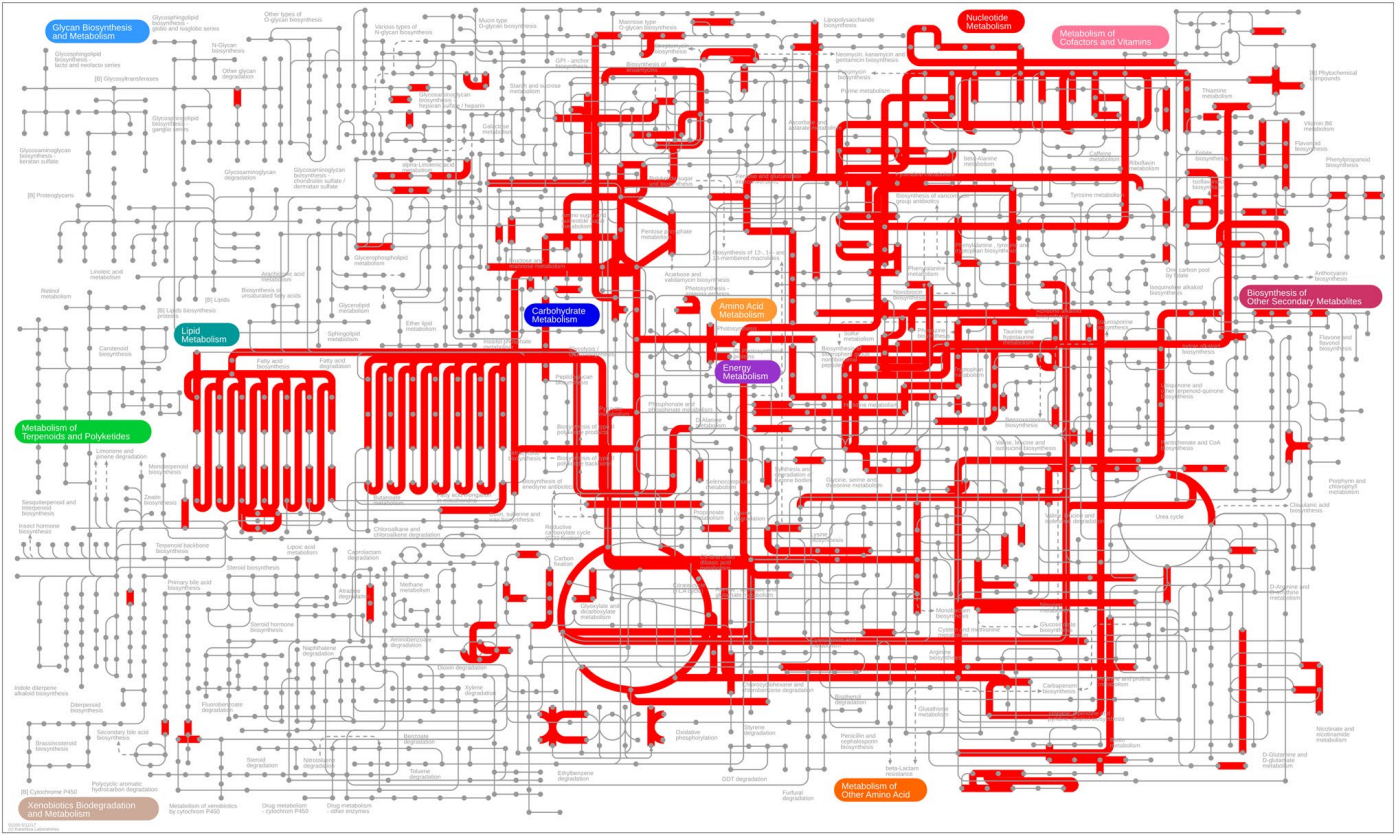
Overview of the metabolic pathways from R6S-5-6. Pathway maps were visualized using iPATH3

A total of 411,483 PacBio reads with the N50 length of 7432 bp were used for genome assembly. Of the four contigs generated under the “meta” option, two were simple repeat sequences and another one was a small assembly artifact of unknown origin, so we eliminated them and used only the remaining contig. Polishing was performed only once because no further corrections were observed. We eventually obtained a complete circular genome with lengths of 3,251,289 bp (759X coverage). The GC content was accounting for 37.68% of the whole-genome. The genome completeness and contamination were determined to be 99.65% and 0.47%, respectively. BUSCO evaluation using flavobacteriales_odb10 showed 100% completeness with no duplicated, fragmented, or missing BUSCOs, revealing high-quality genome can be derived from only using PacBio reads. A total of 3034 genes were predicted, comprising 2940 coding DNA sequences (CDSs), three complete rRNA operons, 33 misc RNAs, 51 tRNAs, and one tmRNA. *gltX* gene, the origin of replication, was located at 710,681–712,192 bp, corresponding to the region where the GC skew converts negative to positive (Fig. 1). The complete genome contained two prophage sequences. There are no CRISPR and virulence genes. Seven genomic islands were identified, of which the GC content decreased sharply between 2.75 and 2.87 M. Figure 2 shows all metabolic pathway of R6S-5-6.

### Confirmation on Taxonomic Status

**Fig. 3:**
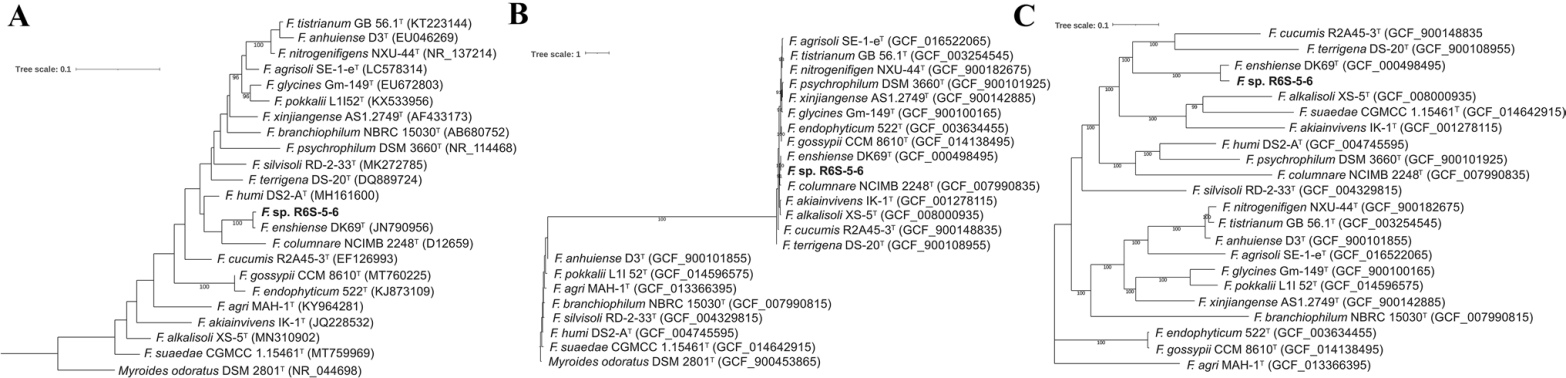
Phylogenetic position of R6S-5-6. The maximum-likelihood trees were constructed from alignment of **A** the full-length of 16S rRNA gene, **B** *gyrB* gene, and **C** 912 single-copy orthologs sequences. Numbers above the branches represent the bootstrapping support values from 1000 replicates (if larger than 80%). Tree scale represents nucleotide substitution per site. *Myroides odoratus* DSM 2801^T^ was used as outgroup in (A) and (B)

To investigate the phylogenetic position of R6S-5-6, we first calculated the 16S rRNA gene sequence similarity against type strains. The sequences of all three 16S rRNA genes in R6S-5-6 were identical, and the closest species was *F. enshiense* DK69^T^ (99.65% similarity) (Fig. 3A). The *gyrB* gene, which was proposed as a phylogenetic marker for genus *Flavobacterium* [16], had a 99.69% protein sequence similarity with that of *F. enshiense* DK69^T^ (Fig. 3B). In the tree based on 912 single-copy orthologs with 21 phylogenetic neighbors, *F. enshiense* DK69^T^ was also the closest species to R6S-5-6 (Fig. 3C). The 16S rRNA gene sequence similarity of strain R6S-5-6 exceeded the species delineation threshold of 98.7%, and protein sequence similarity of the *gyrB* gene also exceeded the intraspecies minimum values of 97.2% [16]. However, with the emergence of high-throughput sequencing technologies, single phylogenetic markers are no longer sufficient for delineating a species. For taxonomic delineation, the 16S rRNA gene sequence similarity should only be used as an initial screening and reliable analysis based on whole-genome sequence is required [17]. Therefore, we first performed TYGS, MiGA, fIDBAC, and GTDB-Tk to assign taxonomic status based on the type strain or representative strain genome. TYGS and GTDB-Tk assigned strain R6S-5-6 as a novel species of the genus *Flavobacterium*. fIDBAC identified strain R6S-5-6 as *F. enshiense* but showed that whole-genome based ANI (gANI) was below the threshold value of 96.5%. MiGA showed an average amino acid identity (AAI) value of 96.58% against *F. enshiense* and concluded that strain R6S-5–6 possibly even belongs to the species *F. enshiense*.

ANI and dDDH, which are in silico genomic metrics, were calculated. The relationship between strain R6S-5-6 and *F. enshiense* DK69^T^ revealed that ANI based on MUMmer (ANIm) value was 91.01%, dDDH value was 69.3% (C.I. 65.4–72.9%) at formula d0, and 40.4% at d4, and GC content differed by 0.05% (Table 1). Since the threshold values for species delineation of ANI and dDDH are 95% ~ 96% and 70%, respectively, the unmet values contradicted the conclusions for the 16S rRNA and *gyrB* gene sequence similarity results. As 95–96% of ANI value is established as ‘the golden standard,’ strain R6S-5-6 is considered a novel species.

**Table 1:** dDDH and ANIm values between R6S-5–6 and related *Flavobacterium* spp

Query genome	Reference genome	dDDH (d0, %)	C.I. (d0, %)	dDDH (d4, %)	C.I. (d4, %)	ANIm value (%)	GC content difference (%)
R6S-5–6	*F. enshiense* DK69^T^	69.3	[65.4–72.9]	40.4	[37.9–43]	91.0	0.05
	*F. akiainvivens* IK-1^T^	13	[10.3–16.3]	19	[16.8–21.3]	85.0	6.12
	*F. tistrianum* GB 56.1^T^	13.3	[10.6–16.6]	20.4	[18.1–22.8]	83.9	3.67
	*F*. *endophyticum* 522^T^	13.4	[10.7–16.7]	19.4	[17.2–21.8]	84.5	1.42
	*F*. *silvisoli* RD-2-33^T^	13.7	[10.9–17]	19.1	[17–21.5]	84.1	1.01
	*F*. *humi* DS2-A^T^	13.8	[11–17.1]	19.4	[17.2–21.8]	82.9	3.06
	*F. branchiophilum* NBRC 15030^T^	12.9	[10.2–16.2]	20.9	[18.6–23.3]	83.7	4.91
	*F. columnare* NCIMB 2248^T^	13.2	[10.5–16.6]	20.5	[18.3–22.9]	84.0	6.41
	*F. alkalisoli* XS-5^T^	13.5	[10.7–16.8]	19	[16.8–21.4]	84.2	0.31
	*F. agri* MAH-1^T^	12.8	[10.1–16.1]	20.2	[18–22.6]	83.2	9.43
	*F. gossypii* CCM 8610^T^	13.6	[10.8–16.9]	18.7	[16.5–21]	84.2	1.21
	*F. pokkalii* L1I52^T^	13.5	[10.7–16.8]	20	[17.8–22.4]	83.9	2.74
	*F. suaedae* CGMCC 1.15461^T^	13.2	[10.5–16.5]	17.6	[15.5–19.9]	83.1	1.87
	*F. agrisoli* SE-1-e^T^	13.3	[10.6–16.6]	20	[17.8–22.4]	84.3	2.85
	*F. glycines* Gm-149^T^	13.5	[10.7–16.8]	19.5	[17.3–21.9]	83.9	3.57
	*F. anhuiense* D3^T^	13.2	[10.4–16.5]	20.5	[18.3–23]	84.0	3.33
	*F. psychrophilum* DSM 3660^T^	13.3	[10.6–16.6]	19.2	[17–21.6]	83.6	5.25
	*F. terrigena* DS-20^T^	13.3	[10.6–16.6]	20.4	[18.2–22.8]	83.7	6.53
	*F. xinjiangense* AS1.2749^T^	13.4	[10.6–16.7]	19.3	[17.1–21.7]	83.6	3.69
	*F. cucumis* R2A45-3^T^	13.9	[11.1–17.2]	19.7	[17.5–22.1]	83.9	4.56
	*F. nitrogenifigens* NXU-44^T^	13.3	[10.5–16.6]	20.1	[17.9–22.5]	84.0	3.59

*C.I.* confidence interval

### Metabolic Pathways of R6S-5–6 Contributing to the Wetland Ecosystem

Some bacteria found in wetlands can contribute positively to the wetland ecosystem in the following ways: (1) purifying wastewater, (2) reducing eutrophication, or (3) promoting plant growth. We highlight genomic properties of R6S-5-6 that have potential for sustaining wetland ecosystems.

**Fig. 4:**
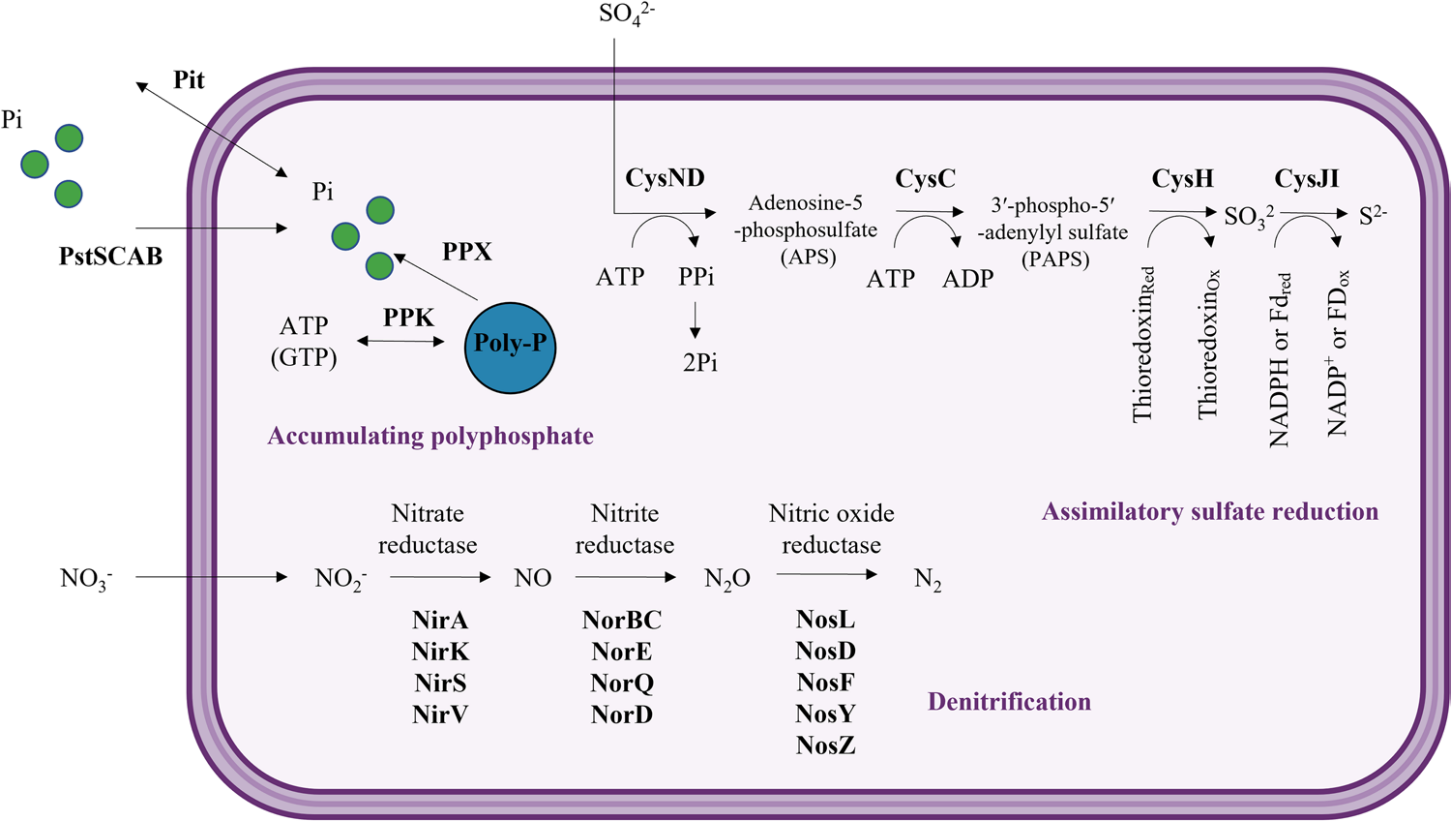
Metabolic pathways of R6S-5-6 related to purification. R6S-5-6 has denitrification, assimilatory sulfate reduction (ASR), and polyphosphate-accumulating process for purifying contaminated water and preventing eutrophication

Wastewater contains nitrogen, phosphorus, and sulfur, which can cause eutrophication. Wetland ecosystems have the ability to purify water, and bacteria have been found to play a major role in this process [18]. In this sense, R6S-5-6 has genes that can contribute to purification (Fig. 4).

### Denitrifying

Denitrifiers in wetlands can control greenhouse effects by removing greenhouse gas N_2_O [19]. R6S-5-6 has the denitrification pathway that is mainly used to remove nitrogen from wastewater [20]. The process consists of four steps: NO_3_^−^ to NO_2_^−^ by nitrate reductase (Nar), NO_2_^−^ to NO by nitrite reductase (Nir), NO to N_2_O by nitric oxide reductase (Nor), and N_2_O to N_2_ by nitrous oxide reductase (Nos) [21]. R6S-5-6 has denitrifying gene clusters which contain Nir (NirA, NirK, NirS, and NirV), Nor (NorBC, NorE, NorQ, and NorD), and Nos (NosL, NosD, NosF, NosY, and NosZ) (Fig. S1).

### Polyphosphate-Accumulating

Excess phosphorus discharged from agriculture and industry adversely affects the ecosystem and causes eutrophication. Accumulating polyphosphate plays an important role in removing soluble phosphorus in wastewater [22]. The ability of bacteria to accumulate poly-p is related to polyphosphate kinases (PPK), exopolyphosphatase (PPX), and phosphate transport systems (pstSCAB and pit) [23]. R6S-5-6 has PPK (EC 2.7.4.1), PPX (EC 3.6.1.11), and phosphate transport (*pstA*, *pstB*, *pstC*, *pstS*, and *pitA*).

### Sulfate-Reducing

Sulfate present in wastewater is removed through assimilatory sulfate reduction (ASR) pathway [24]. Sulfate is reduced to sulfide through the ASR metabolic pathway that proceeds over four steps: SO_4_^2−^ → adenosine-5-phosphosulfate (APS) → 3′-phospho-5′-adenylyl sulfate (PAPS) → SO_3_^2−^ → S^2−^ [25]. R6S-5-6 has the genes *cysN*/*cysD*, *cysD*, *cysH*, and *cysJ*/*cysI* encoding enzymes associated with the ASR pathway (Fig. S2).

### Other Organic Pollutants

Analysis of the genome using dbCAN showed 72 genes encoding for CAZymes, including glycoside hydrolases (GHs) (*n* = 13), glycosyltransferases (GTs) (*n* = 44), carbohydrate esterases (CEs) (*n* = 12), carbohydrate-binding modules (CBMs) (*n* = 1), and auxiliary activities (AAs) (*n* = 2). GTs are most abundant, accounting for 61% of the total number of predicted CAZymes. GT families are involved in the biosynthesis of compounds such as oligosaccharides, polysaccharides, and glycoconjugates. The role of this enzyme is important in that it converts potential toxic organic pollutants into low-molecular-weight intermediates or harmless terminal products [26]. As a related study, GT families are known to help remove total dissolved solids (TDS) and chemical oxygen demand (COD) loading macronutrients [27].

### R6S-5–6 Affects the Plant Ecosystem in Wetlands

Recent studies have shown that some *Flavobacterium* spp. have a positive effect on plants. Plant growth-promoting bacteria (PGPB) contribute to plants as follows: (1) storage of essential nutrients for plants, (2) promotion of plant growth, and (3) plant protection. Considering this, several genes of R6S-5-6 emphasize some useful effects on plant ecology in wetlands.

### Storage of Essential Nutrients for Plant Growth

Nitrogen and phosphorus are essential nutrients for plants but they are limited in the rhizosphere [28]. Nitrogen-fixing bacteria have nitrogenase genes, mainly *nif* genes [29]. In this sense, R6S-5-6 contains Nif gene (*nifU*), nitrogen fixation regulation protein (FixK), and Global nitrogen regulator (NtcA) associated with nitrogen-fixing.

Alkaline phosphatase (ALP) converts organic phosphorus to available phosphorus for plants [30]. However, ALP is mainly produced by soil microbes [31]. R6S-5-6 has an ALP (EC 3.1.3.1), through which organic phosphorus is mineralized. The solubilization of inorganic phosphorus occurs by the action of organic acids such as gluconic acid [32]. PyrroloQuinoline Quinone (PQQ) catalyzes gluconic acid [33]. R6S-5-6 has *pqqL* gene predicted to be related to PQQ production [34].

### Promotion of Plant Growth

Auxin is a plant hormone that promotes plant growth and development [35]. Indole-3-acetic acid (IAA), which is a representative auxin, is synthesized with tryptophan as a precursor [36]. R6S-5-6 has a tryptophan (trp) biosynthesis pathway, which contains trp synthase alpha chain (EC 4.2.1.20), trp synthase beta chain (EC 4.2.1.20), anthranilate phosphoribosyltransferase (EC 2.4.2.18), and phosphoribosylanthranilate isomerase (EC 5.3.1.24) (Fig. S3).

Bacteria with 1-aminocyclopropane-1-carboxylate (ACC) deaminase absorb some of the ACC synthesized by plants. ACC is decomposed by ACC deaminase and easily metabolized in bacteria. Lowering ACC in plants reduces the amount of ethylene. This prevents the effect of stress ethylene generated in stressful situations and helps plants grow [36]. R6S-5–6 has ACC deaminase (EC 3.5.99.7).

### Plant Protection

The nonribosomal peptide synthetase and polyketide synthase (NRPS-PKS) gene cluster of *Flavobacterium* isolated from plants is known to be involved in the biosynthesis of compounds that inhibit plant pathogens [37]. R6S-5-6 has twenty NRPS-PKS gene clusters, of which it has the highest homogeneity with the NRPS-PKS gene cluster of *Aquimarina* sp. BL5 at 40%.

The antiSMASH results indicated that R6S-5-6 has gene clusters for flexirubin and APE Vf. Flexirubin which are widely distributed in the genus *Flavobacterium*. They are pigments that form a yellow colony and are composed of a 2–5 dialkylresorcinol (DAR) with a non-isoprenoid aryl-polyene carboxylic acid ester [38, 39]. Although their ecological roles are unknown, DAR compounds are known to have antibacterial properties in *Pseudomonas* sp. [40].

Cell wall dissolving enzymes produced by PGPB can break down the cell walls of plant pathogens. R6S-5-6 has cellulase (EC 3.2.1.4), glucanase (EC 3.2.1.21), and β-1,3-glucanase (EC 3.2.1.39) which are known to contribute to plant defense by degrading the cell walls of plant pathogens [41, 42].

## Discussion

In wetlands where various living things are in harmony, bacteria play a significant role in maintaining the ecosystem, including purifying pollutants and promoting plant growth. However, bacteria and their genomes isolated from wetlands have been scarcely reported. Therefore, in this study, we investigated the genomic characteristics of R6S-5-6 which was isolated from Ungok Wetland by conducting de novo assembly.

We performed several analyses to confirm the taxonomic status of R6S-5-6. Interestingly, all metrics except for ANI concluded that R6S-5-6 belongs to *F. enshiense*. In this regard, there have been studies that ANI cannot perfectly reflect the evolutionary distance of all members of bacteria, including that the threshold value for ANI was inflated by using highly redundant genomes [43] and that cutoff values should be lowered in some species such as *Variovorax paradoxus* and *Stenotrophomonas maltophilia* [44]. Also, according to a recent paper from *Donovan* et al. [45], intraspecies ANI values are expected to approach 90%, a 10% diameter cluster with the representative genome. Since there is only one strain in *F. enshiense*, and it was isolated from the waste liquid treatment facility of the pharmaceutical company, we think it should be discreet in proposing R6S-5-6 as a novel species. Since pharmaceutical waste has mutagenicity and genotoxicity [46], it is thought that many synonymous mutations may have occurred at low ANI value compared to AAI value. Taken together, we cautiously suggest that R6S-5-6 is the strain within the species *F. enshiense* located at the species boundary with type strain DK69. We expect that the phylogenetic position will be changed more precisely if the study of *F. enshiense* continues and additional strains are reported, as in the case where the species *Lactobacillus gasseri* was recently divided into *L. gasseri* and *Lactobacillus paragasseri* due to intraspecies heterogeneity [47]. In addition, chemotaxonomic information that can be derived from physiological and biochemical experiments will support the taxonomic status confirmation more reliably. Therefore, fatty acid analysis, biochemical API testing, and microscopic morphological observation should also be conducted in future studies.

To predict the role of *F. enshiense* R6S-5-6 in the wetland ecosystem, we looked into the pathways related to the purification of wetlands and plant growth. R6S-5-6 has genes involved in denitrifying, polyphosphate-accumulating, and assimilatory sulfate-reducing pathways for wastewater purification. Regarding plant growth in wetland ecosystems, R6S-5-6 has genes that store nitrogen and phosphorus for plant use. R6S-5-6 also has genes encoding tryptophan biosynthesis-related enzymes and ACC deaminase that inhibit plant pathogens. These results suggest that *F. enshiense* R6S-5-6 has the potential to play a beneficial role in the wetland community.

## Conclusion

In this study, we analyzed the role of *F. enshiense* R6S-5-6 in the wetland ecosystem. Unlike previous investigations of *Flavobacterium* spp., which mainly focused on pathogenic characteristics, we provided information on how R6S-5-6 is involved in the circulation of wetland and specific pathways in plant ecosystems. The genomic characteristics of R6S-5-6 investigated in this study will broaden understanding of species *F. enshiense* where only one strain has been reported.

## Supplementary Information

Below is the link to the electronic supplementary material.

Supplementary file1 (PDF 157 kb)

## Data Availability

The complete genome sequence of *F. enshiense* R6S-5-6 has been deposited in GenBank under the accession number CP090376.
