# Supplementary material for: The Role of Flavobacterium enshiense R6S-5-6 in the Wetland Ecosystem Revealed by Whole-Genome Analysis
Source: Curr Microbiol. 2023 Jan 21;80(2):83. doi: 10.1007/s00284-022-03157-0 (PMC9867689; doi:10.1007/s00284-022-03157-0)
Supplement: Supplementary file 1 — Supplementary file1 (PDF 157 kb) [file 284_2022_3157_MOESM1_ESM.pdf]

SUPPLEMENTARY MATERIAL

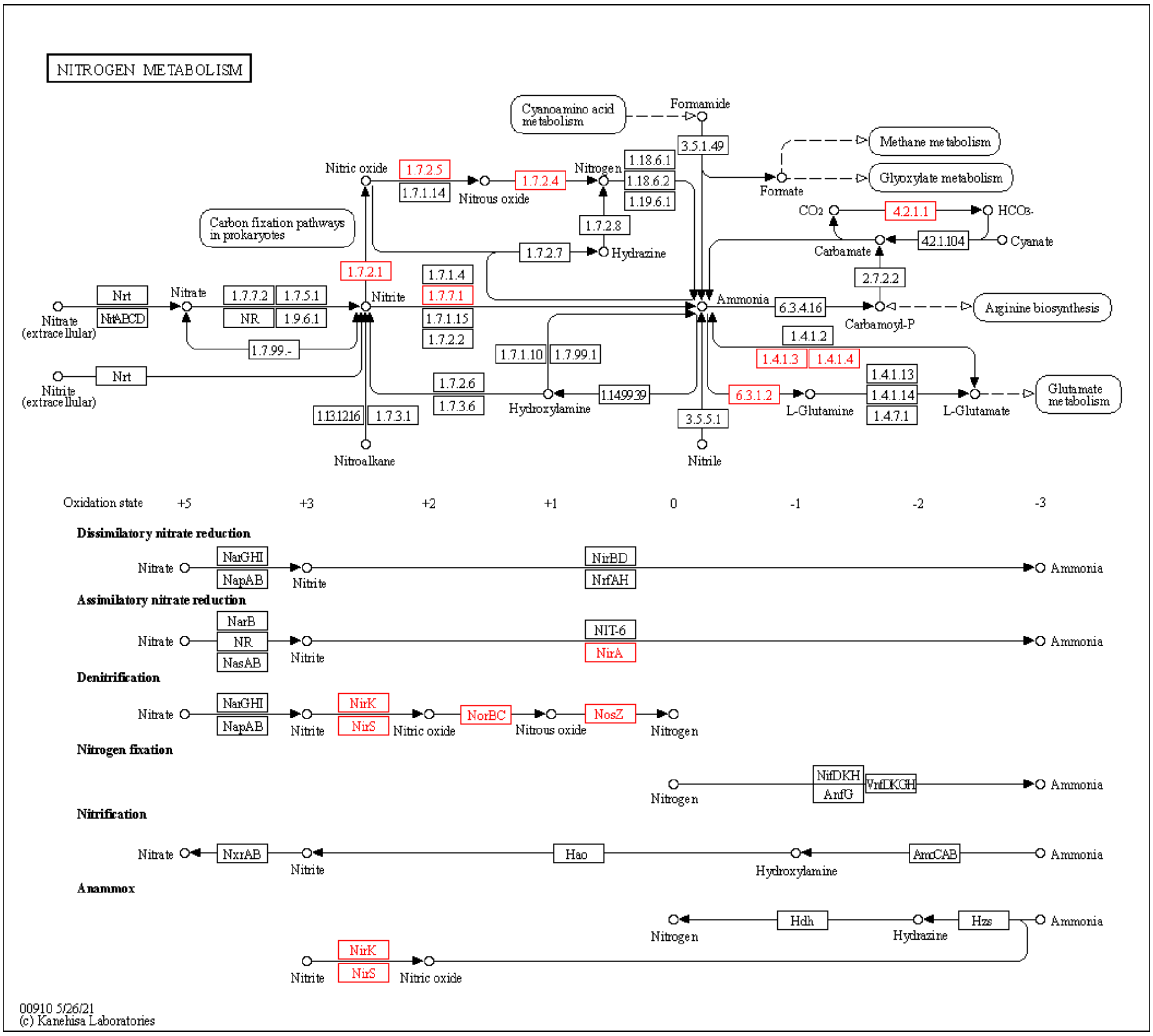

Supplemental Figure 1. Nitrogen metabolism from R6S-5-6. Red letter is an enzyme and gene of the R6S-5-6. In particular, R6S-5-6 has a denitrification process in the nitrogen metabolic pathway.

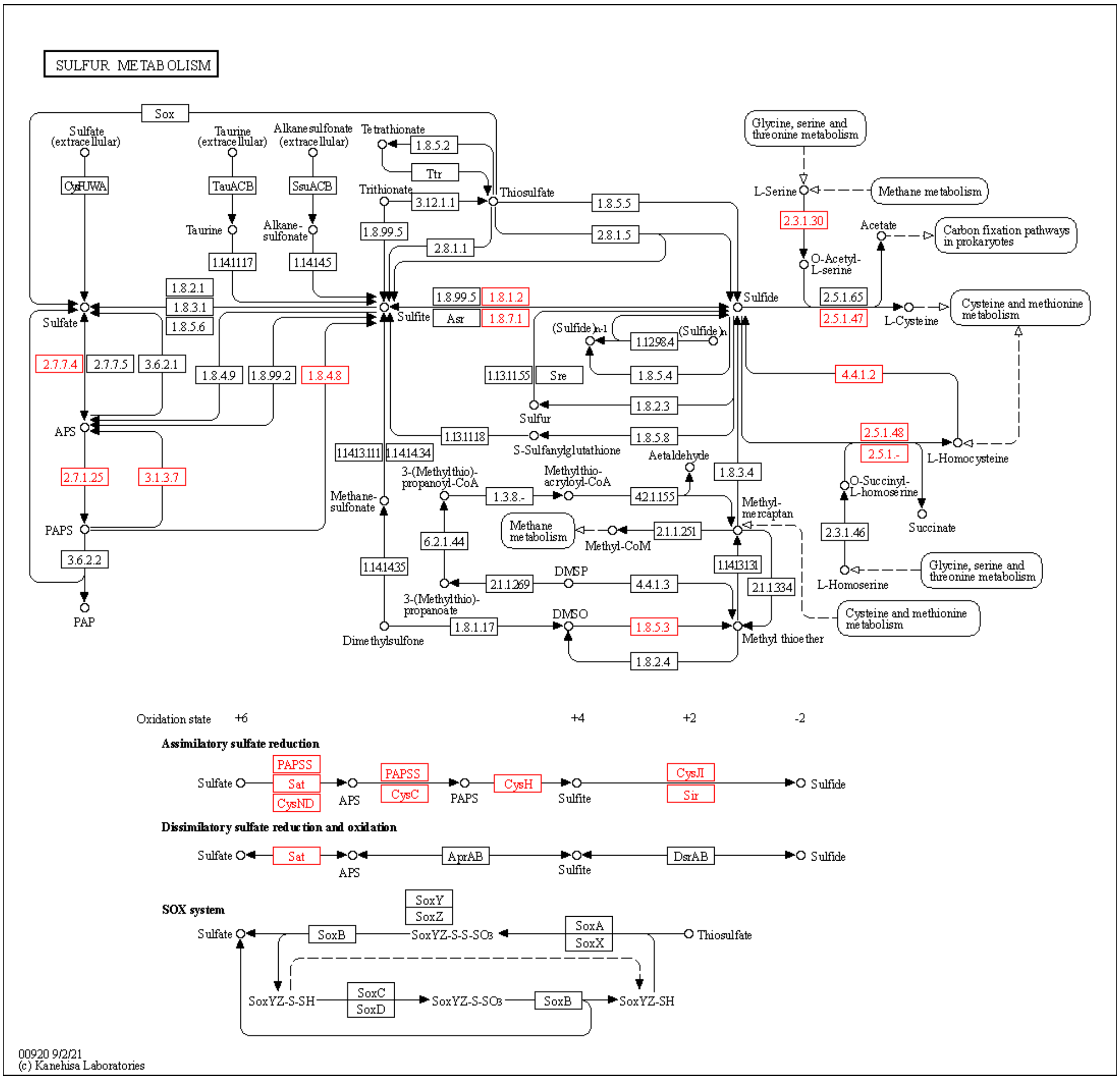

Supplemental Figure 2. Sulfur metabolism from R6S-5-6. Red letter is an enzyme and gene of the R6S-5-6. In particular, R6S-5-6 has an assimilatory sulfate reduction pathway (ASR) in the sulfur metabolic pathway.

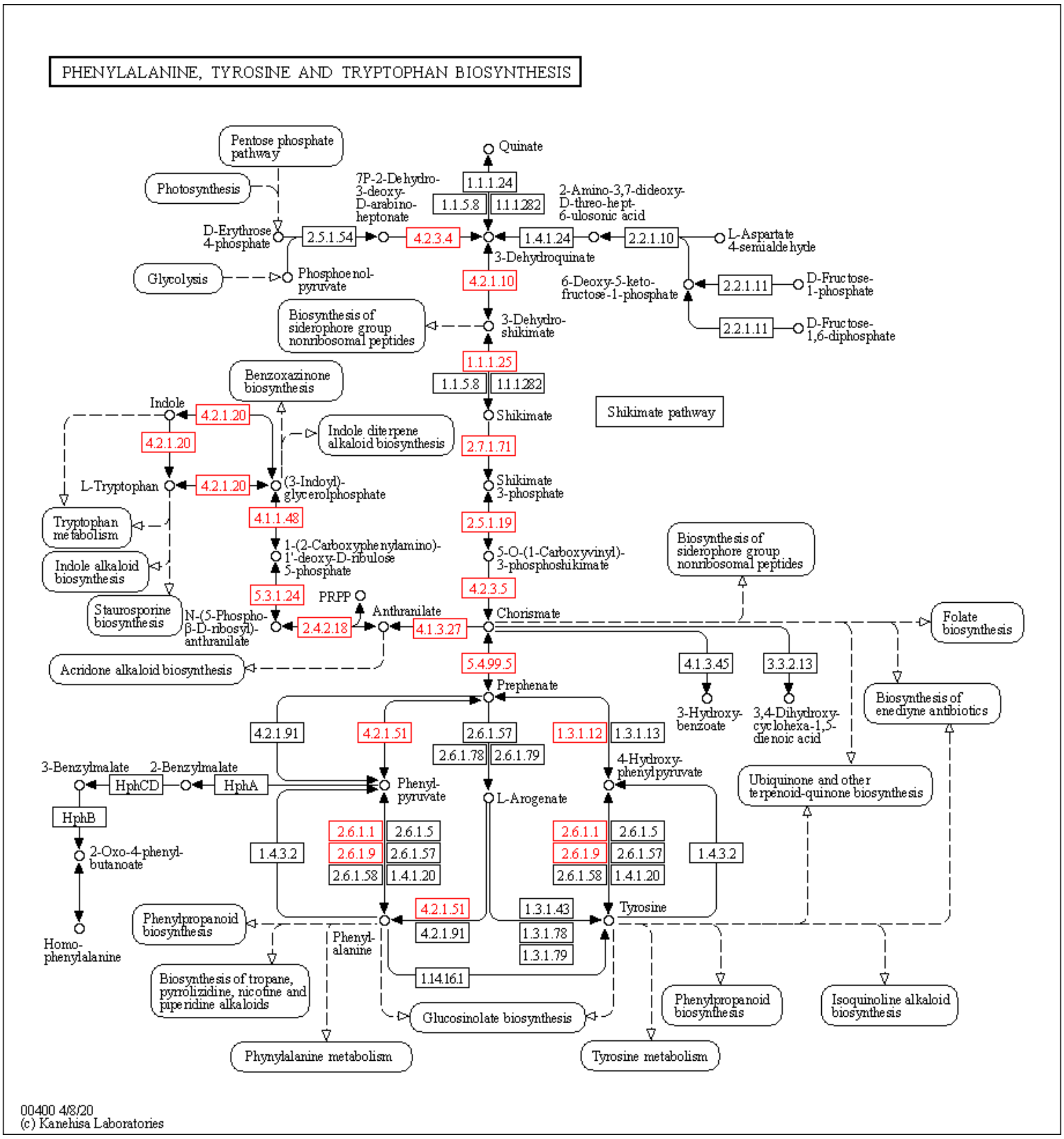

Supplemental Figure 3. Tryptophan biosynthesis process from R6S-5-6. Red letter is an enzyme and gene of the R6S-5-6. In particular, R6S-5-6 has Tryptophan biosynthesis process to produce IAA.
